# Diagnostic Accuracy in Teleneurological Stroke Consultations

**DOI:** 10.3390/jcm10061170

**Published:** 2021-03-11

**Authors:** Jordi Kühne Escolà, Simon Nagel, Christina Verez Sola, Eva Doroszewski, Hannah Jaschonek, Alexander Gutschalk, Christoph Gumbinger, Jan C. Purrucker

**Affiliations:** Department of Neurology, Heidelberg University Hospital, 69120 Heidelberg, Germany; jordi.kuehneescola@gmail.com (J.K.E.); simon.nagel@med.uni-heidelberg.de (S.N.); christinaverez@yahoo.de (C.V.S.); eva.doroszewski@med.uni-heidelberg.de (E.D.); hannah.jaschonek@med.uni-heidelberg.de (H.J.); alexander.gutschalk@med.uni-heidelberg.de (A.G.); christoph.gumbinger@med.uni-heidelberg.de (C.G.)

**Keywords:** cerebral stroke, cerebrovascular disease, diagnostics, telemedicine, teleneurology

## Abstract

Background: The accuracy of diagnosing acute cerebrovascular disease via a teleneurology service and the characteristics of misdiagnosed patients are insufficiently known. Methods: A random sample (*n* = 1500) of all teleneurological consultations conducted between July 2015 and December 2017 was screened. Teleneurological diagnosis and hospital discharge diagnosis were compared. Diagnoses were then grouped into two main categories: cerebrovascular disease (CVD) and noncerebrovascular disease. Test characteristics were calculated. Results: Out of 1078 consultations, 52% (*n* = 561) had a final diagnosis of CVD. Patients with CVD could be accurately identified via teleneurological consultation (sensitivity 95.2%, 95% CI 93.2–96.8), but we observed a tendency towards false-positive diagnosis (specificity 77.4%, 95% CI 73.6–80.8). Characteristics of patients with a false-negative CVD diagnosis were similar to those of patients with a true-positive diagnosis, but patients with a false-negative CVD diagnosis had ischemic heart disease less frequently. In retrospect, one patient would have been considered a candidate for intravenous thrombolysis (0.2%). Conclusions: Teleneurological consultations are accurate for identifying patients with CVD, and there is a very low rate of missed candidates for thrombolysis. Apart from a lower prevalence of ischemic heart disease, characteristics of “stroke chameleons” were similar to those of correctly identified CVD patients.

## 1. Introduction

Intravenous thrombolysis (IVT) and endovascular therapy (EVT) have become cornerstones of acute stroke management and can significantly improve patients’ functional outcomes [1,2]. However, these treatment options are highly time-sensitive and require neurological expertise, which is not available at all hospitals, especially in rural areas. The use of telemedicine has been among the potential solutions to overcome the lack of neurological expertise in stroke care by enabling remote consultations for patients admitted to local hospitals, whereby long transfers of patients in a time-critical condition can be avoided [3,4,5].

Rapidly expanding telestroke services have facilitated access to neurological expertise in remote areas and provided greater availability of safe and effective thrombolytic treatment [6]. However, data on the diagnostic accuracy of teleneurological stroke consultations are still sparse. A previous study reported poorer diagnostic accuracy for nonstroke cases than for stroke cases, for which initial teleneurological consultations were conducted between 2008 and 2009 [7].

A delayed or missed diagnosis of stroke (false-negative, stroke chameleon) can preclude acute treatment, making stroke management particularly vulnerable to diagnostic error [8]. This issue also affects false-positive stroke diagnosis (stroke mimic), which may expose patients to unnecessary diagnostics and therapy, waste limited resources, and cause additional costs [9] but is associated with minimal concerns for patient safety [10].

Hence, the purpose of this study is to provide a better understanding of the diagnostic accuracy and misdiagnosis in teleneurological stroke consultations. Our main objective was to characterize the accuracy of diagnosing acute cerebrovascular disease via teleneurology in a large teleneurology network in Germany. We furthermore aimed to explore the characteristics of misdiagnosed patients with a focus on stroke chameleons.

## 2. Methods

### 2.1. Study Design and Setting

The Heidelberg University Hospital provides a 24-h teleneurology service 7 days a week to seven regional sites. After being primarily admitted to a spoke clinic, patients with suspected stroke are evaluated via a two-way real-time audiovisual communication system, while access to imaging data is provided by teleradiology. Two of seven sites are small regional neurology departments that offer permanent local neurological expertise, also beyond the field of stroke, during regular working hours. At the five other sites, residents and consultants from the internal medicine departments handle the acute patients with teleneurological support during nonworking hours. During emergency consultations, initial diagnosis is made by the teleneurological consultant, including recommendations on further timely diagnostic and therapeutic procedures. After the consultation, most patients remain at the local primary stroke center (PSC), while some are acutely transferred to the hub if the teleneurologist deems it necessary. All stroke patients are seen daily by a local neurologist, who makes further assessments and the final diagnosis.

We conducted a retrospective analysis of a random sample (*n* = 1500) of all teleneurological consultations between July 2015 and December 2017. The sample size was chosen based on the aim of screening a set of randomly selected 25% of teleneurological consultations from the given period, and the resulting number was rounded down to the next hundred. The study was approved by the ethics committee of the Medical Faculty of the University of Heidelberg (S-007/2018).

### 2.2. Data Acquisition and Diagnostic Categories

Teleneurological consultation records and final discharge reports were extracted from the digital hospital archive. Demographic characteristics, medical history, premorbid modified Rankin scale score (pmRS, ranging from 0 (no disability) to 5 (severe disability)), National Institutes of Health stroke scale score (NIHSS) at admission, imaging data, and information on acute and postacute stroke treatment were registered. Initial and final diagnoses were recorded as defined by the teleneurological consultant and hospital discharge diagnoses, respectively. Diagnoses were then grouped into two main categories: (I) cerebrovascular disease, defined as a diagnosis of ischemic stroke (IS), transient ischemic attack (TIA), intracerebral hemorrhage (ICH), subarachnoid hemorrhage (SAH), or cerebral venous sinus thrombosis (CVST), and (II) noncerebrovascular disease diagnoses. Data were processed using Microsoft Excel (Microsoft Corp., Redmond, WA, USA).

### 2.3. Statistical Analysis

Initial teleneurological diagnosis was compared with the reference standard of final diagnosis and classified as true positive, true negative, false positive, or false negative for the presence of cerebrovascular disease. Sensitivity, specificity, positive predictive value (PPV), negative predictive value (NPV), and area under the receiver operating characteristic curve (AUC) were computed as measures of diagnostic accuracy. Descriptive statistics were used to further characterize patients who were misdiagnosed via teleneurology. Continuous data are described as mean (SD) or median (interquartile range), while categorical data are presented as absolute and relative frequencies (counts and percentages). The Kolmogorov–Smirnov test was used to ascertain the distribution of the data. Baseline demographic and clinical characteristics of patients with a false-positive and a false-negative diagnosis of cerebrovascular disease via teleneurology were compared with those characteristics of patients with a true-positive diagnosis. The Fisher exact test or nonparametric Mann–Whitney U test was used according to the level of measurement. All statistical tests were two-sided, and *p* values of <0.05 were considered statistically significant. No adjustment was made for multiple testing. Statistical analysis was performed using versions 26 and 27 of SPSS (IBM Corp., Armonk, NY, USA).

## 3. Results

**Figure 1:**
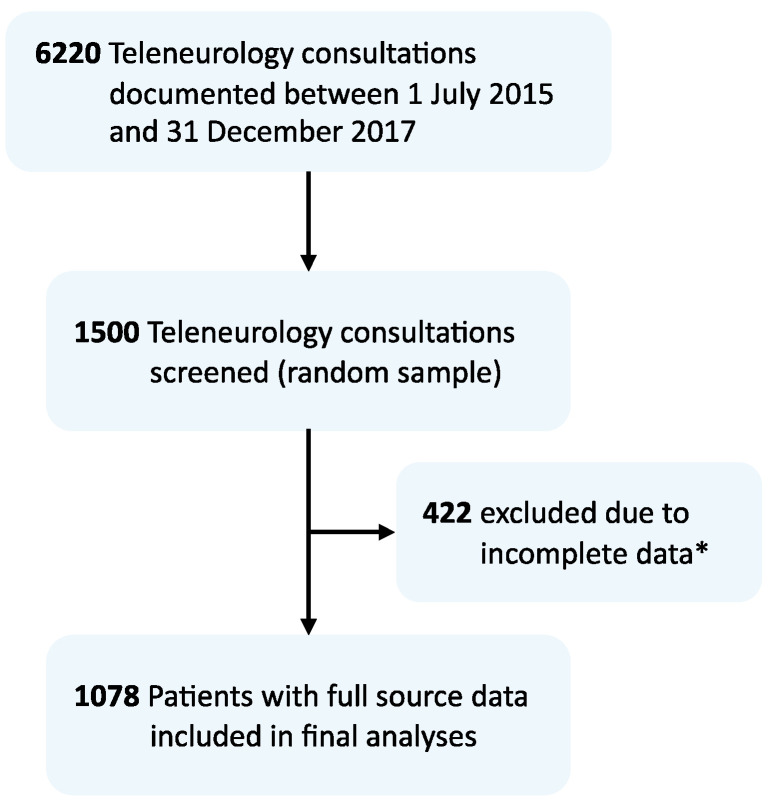
Flow chart of study population. * Of the 422 consultations excluded from analysis, *n* = 164 patients were excluded due to incomplete discharge data transfer to hub. In *n* = 228 patients, discharge data sets were incomplete or missing due to patient transfer to third-party CSC, discharge against medical advice, errors linking archived documents to teleneurological consultations, or changes in hospital information system documentation standards (1 center). Another *n* = 30 consultations were ultimately canceled or performed with no written standardized documentation.

**Figure 2:**
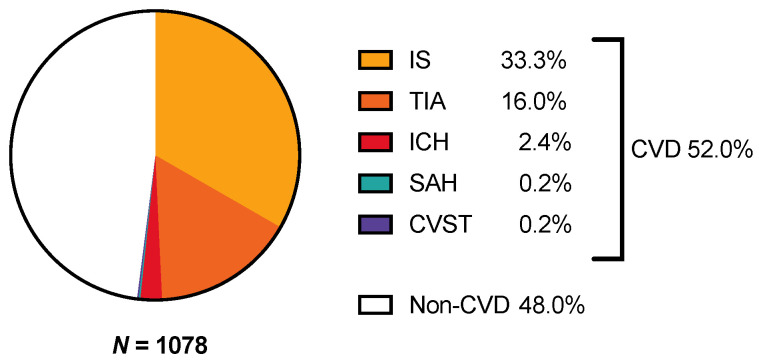
Final CVD diagnoses. Abbreviations: CVD, cerebrovascular disease; CVST, cerebral venous sinus thrombosis; ICH, intracerebral hemorrhage; IS, ischemic stroke; SAH, subarachnoid hemorrhage; TIA, transient ischemic attack.

Of the 6220 teleneurological consultations conducted between 2015 and 2017, a random sample of *n* = 1500 (24.1%) was selected (see Figure 1). Full source data were available for *n* = 1078 consultations. More than half of the patients (52%) had a final diagnosis of cerebrovascular disease (see Figure 2), including ischemic stroke (*n* = 359), TIA (*n* = 172), ICH (*n* = 26), SAH (*n* = 2), and CVST (*n* = 2). Noncerebrovascular final diagnoses were common (48%), and seizures were the most frequent among them (*n* = 89). In 7% of all the teleneurological consultations (*n* = 72), the underlying etiology remained uncertain at discharge.

### 3.1. Diagnostic Accuracy

**Figure 3:**
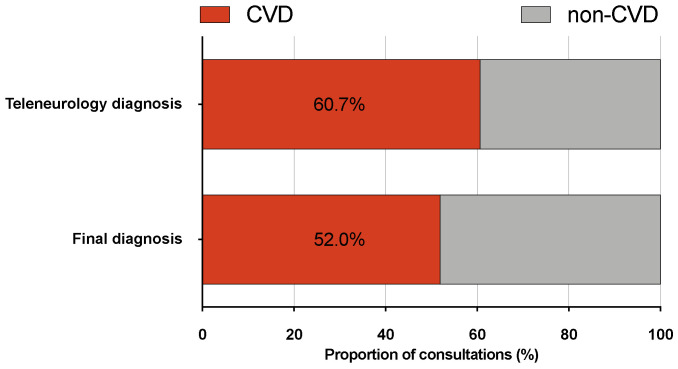
Frequency of cerebrovascular disease vs. other diagnoses after initial teleneurological consultation and at discharge. CVD, cerebrovascular disease.

Cerebrovascular disease could be accurately identified in patients via teleneurology (sensitivity 95.2%, 95% CI 93.2–96.8; NPV 93.6%, 95% CI 91.0–95.7) but showed a tendency towards false-positive diagnosis (specificity 77.4%, 95% CI 73.6–80.8; PPV 81.7, 95% CI 78.6–84.5). AUC was 0.860 (95% CI 0.836–0.884) (see Figure 3 for shift of diagnoses).

### 3.2. Misdiagnosis

Several differences were observed between misdiagnosed patients and those with correctly diagnosed cerebrovascular disease (see Table 1 and Appendix A Table A1 and Table A2 for the full list of descriptive comparisons). Patients with a false-positive diagnosis were younger than patients with a true-positive diagnosis (median age 73.2 years, IQR 60.7–81.0 vs. 76.4, 66.1–82.8; *p* = 0.012), had a lower prevalence of arterial hypertension (51.3% vs. 71.9%; *p* < 0.001), and showed a higher prevalence of prior seizures (7.5% vs. 2.2%; *p* = 0.007). Median NIHSS was lower than it was for correctly diagnosed patients (median NIHSS 1, IQR 0–3 vs. 3, 1–7; *p* < 0.001). Baseline characteristics of patients with a false-negative diagnosis of cerebrovascular disease via teleneurology were more like those of patients with a true-positive diagnosis, despite a lower prevalence of ischemic heart disease (3.7% vs. 18.9%; *p* = 0.042).

### 3.3. False-Positive Diagnosis—Stroke Mimics

**Figure A1:**
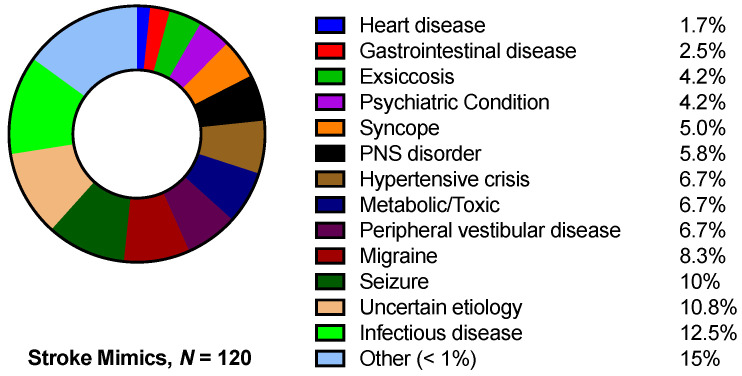
Types of stroke mimics. PNS, peripheral nervous system.

Noncerebrovascular final diagnoses were found in 120 out of 654 patients (18.3%) who had received a diagnosis of cerebrovascular disease via teleneurological consultation. Five of these patients (4%) were transferred to our comprehensive stroke center (CSC) for further diagnostic evaluation, and one patient underwent IVT (0.2%). Overall, final diagnoses were heterogeneous, including more than 30 different stroke mimics, among which infectious diseases (*n* = 15) and seizures (*n* = 12) were observed most frequently (see Figure A1). The etiology remained uncertain at discharge in *n* = 13 patients.

### 3.4. False-Negative Diagnosis—Stroke Chameleons

**Figure 4:**
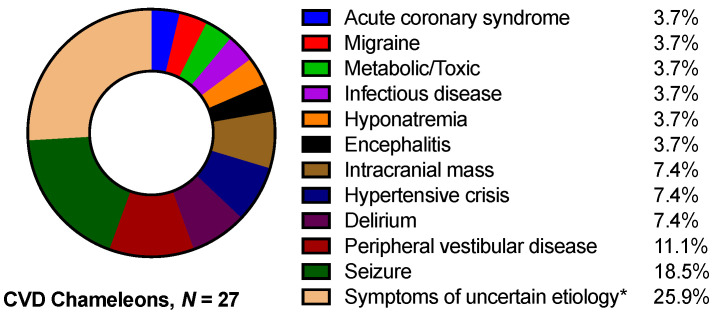
Types of CVD chameleons. * Symptoms of uncertain etiology were purely sensory (*n* = 4), unsteadiness (*n* = 2), and altered level of consciousness (*n* = 1).

Out of the 561 patients with a final diagnosis of cerebrovascular disease, 4.8% (ischemic stroke, *n* = 15; TIA, *n* = 12) had received a noncerebrovascular teleneurological diagnosis (stroke chameleon). Out of the 27 patients, 26 patients were primarily admitted to the local PSC (general ward, *n* = 6; intermediate care ward or stroke unit, *n* = 19; and intensive care unit, *n* = 1) and 1 patient was transferred for MRI imaging and subsequently admitted to the CSC stroke unit. Among the most common stroke chameleons (see Figure 4) were seizures (*n* = 5) and peripheral vestibular disease (*n* = 3). However, in *n* = 7 cases, a potentially underlying etiology could not be further defined in the initial teleneurological consultation. In retrospect, *n* = 3 patients with acute ischemic stroke presented within 4.5 h after symptom onset and had no known contraindication for intravenous thrombolysis (IVT). Two of these patients presented nondisabling symptoms (NIHSS < 2) and therefore would probably not have been considered as candidates for receiving IVT, which suggests one potentially missed thrombolysis due to underdiagnosis (0.2%).

## 4. Discussion

Among over 1000 randomly selected patients who received teleneurological consultations in our large teleneurology network in Germany, a diagnosis of cerebrovascular disease was missed in only 27 out of 561 cases (4.8%), including one potentially missed opportunity for treatment with intravenous thrombolysis (0.2%). Of the 517 patients with a final diagnosis other than cerebrovascular disease, one patient (0.2%) inadvertently received thrombolytic treatment. The sensitivity for diagnosis of cerebrovascular disease via teleneurology was over 95% (95% CI, 93.2–96.8), and the overall diagnostic accuracy was very good (AUC, 0.860; 95% CI, 0.836–0.884).

However, we observed a tendency towards false-positive diagnosis. Nearly half of the patients (48%) were discharged with a noncerebrovascular final diagnosis, and in one-fifth (18%) of the patients with a suspected diagnosis of cerebrovascular disease via teleneurological consultation, the final diagnosis was different. Conditions that frequently mimicked cerebrovascular disease in teleneurological consultations were systemic infections, seizures, and migraines, all of which have been previously described as common stroke mimics in conventional in-person settings [10,11,12]. However, diagnoses were heterogeneous with over 30 different conditions. Several clinical and demographic features have been found to be associated with stroke mimics [13] and have been evaluated in the context of telestroke [14,15]. In line with these findings, we observed that patients who were overdiagnosed via teleneurological consultation were younger, had a lower prevalence of arterial hypertension, had a higher prevalence of prior seizures, and presented a lower median NIHSS than patients with correctly diagnosed cerebrovascular disease. However, we did not find differences for other vascular risk factors, such as atrial fibrillation.

We also investigated demographic characteristics of patients with a final diagnosis of cerebrovascular disease who were misdiagnosed via teleneurological consultation. Except for a lower prevalence of ischemic heart disease, we did not observe differences between true-positive and false-negative initial diagnoses of cerebrovascular disease. In part, these findings are not in line with previous work that found patients with stroke chameleons to be younger and present fewer vascular risk factors [16]. This suggests that, in patients with such characteristics, teleneurological consultation may not have been sought in the first place. Although our sample size of underdiagnosed patients was relatively small, this should raise awareness for missed stroke diagnoses in patients of younger age with a lower vascular risk profile. Data on stroke chameleons in teleneurology are scarce. Whether and how underlying circumstances differ from traditional settings remains unclear, as do the associated outcomes, and further investigation is required.

Given that the main focus of our teleneurology network is to provide neurological expertise in time-sensitive emergencies such as acute ischemic stroke, the rate of 48% of noncerebrovascular final diagnoses appears surprisingly high. Overall, however, stroke mimic rates in acute stroke evaluations of up to 24–44% have been reported from emergency departments [13,17] and telestroke networks [14]. The relatively high rate compared to other networks may be influenced by the fact that two of the primary stroke centers also run a regional neurology department and some acute nonstroke patients were assessed via the teleneurology service. However, low emergency department thresholds to request consultation via our network are the most likely cause. Furthermore, as the burden of a missed stroke outweighs the initially higher diagnostic and treatment standards due to admissions to a monitored ward/stroke unit, a higher sensitivity is considered more relevant.

Factors that are responsible for misdiagnosis in conventional stroke consultations probably apply to teleneurological settings as well. Some clinical features such as transient or unspecific symptoms like dizziness and vertigo can be difficult to evaluate, and making a correct diagnosis becomes even more challenging if the patient is assessed via video consultation only. In addition, there might be systemic sources of error specifically related to teleneurological consultations. Communication and data transfer between spoke clinics and the hub can be flawed, resulting in incomplete or erroneous transmission of information and ultimately leading to misdiagnosis. In cases of unclear clinical presentation, potential solutions to minimize diagnostic error could include screening tools such as the TeleStroke mimic (TM) score [15], advanced imaging during acute phase (e.g., multimodal CT or MRI), or technical improvements such as the use of video head impulse test (vHIT) systems [18]. The implementation of modern data infrastructures such as cloud-based data sharing and digital patient files could further prevent loss of information and thus lead to an improvement of diagnostic accuracy in teleneurological stroke consultations. The use of artificial intelligence is currently planned for investigation in prospective studies.

Our study has limitations. Data were obtained in a single telemedical network. Additionally, data were collected during clinical routine and may be inaccurate or incomplete in some cases. Some data on cardiovascular risk factors such as smoking and alcohol consumption were missing in a significant number of cases. We did not systematically record specific stroke symptoms, despite their important role in distinguishing cerebrovascular disease from stroke mimics and chameleons. Although we found a high diagnostic accuracy for identifying patients with cerebrovascular disease in general, diagnostic error may be significantly higher among specific subgroups, such as patients with mild and/or transient symptoms [19]. Despite the relatively large sample of consultations, the number of patients in the false-negative group remained small. Furthermore, telehealth is prone to supervisor bias, which we tried to minimize by picking a random sample of all teleneurological consultations conducted during the study period. We took the final diagnosis as a reference standard; however, incorrect diagnoses may not have been revised properly, in part due to the anchoring on the initial teleneurological consultation. As a result, rates of misdiagnosed patients may be underestimated.

Nonetheless, our study has several strengths. Although diagnostic accuracy is recognized as an important indicator of quality, final diagnoses are often not documented in teleneurology networks [6]. We were able to analyze data from seven regional stroke centers and one academic tertiary care center in a large geographic area in southwestern Germany. As incomplete documentation was the only criterion for exclusion, discharge documentation was available for all included patients. Although we report data from a single network, our findings give insight into the real-world use of teleneurology and contribute to the globally growing experience from different telehealth services.

## 5. Conclusions

In conclusion, we have shown that teleneurological consultations are accurate in identifying patients with cerebrovascular disease and characterized patients who were misdiagnosed via teleneurology. However, factors that are associated with misdiagnosis in teleneurology require further investigation. In addition, future studies should focus on critical decision-making processes during the preclinical phase and in emergency departments at the local hospitals, where stroke diagnoses could be missed at substantial rates [19,20,21]. Data from prospective registries will be needed to provide a more holistic understanding of diagnostic accuracy and misdiagnosis in teleneurology networks.

## Figures and Tables

**Table 1:** Patient characteristics categorized according to correctness of teleneurological diagnosis of cerebrovascular disease based on *n* = 1078 consultations.

	True Positive *n* = 534	True Negative *n* = 397	False Positive *n* = 120	False Negative *n* = 27
Age	76.4 (66.1–82.8)	66.2 (50.2–78.1)	73.2 (60.7–81.0)	78.7 (61.2–85.4)
Female sex	254 (47.6)	212 (53.4)	59 (49.2)	13 (48.1)
Medical history				
Arterial hypertension	384 (71.9)	161 (40.6)	61 (51.3)	20 (74.1)
Atrial fibrillation	120 (22.5)	48 (12.1)	27 (22.5)	5 (18.5)
Diabetes mellitus	157 (29.5)	50 (12.6)	26 (21.7)	7 (25.9)
Hyperlipidemia	151 (28.3)	42 (10.6)	26 (21.7)	5 (18.5)
Intracranial hemorrhage	11 (2.1)	17 (4.3)	4 (3.3)	–
Ischemic heart disease	101 (18.9)	35 (8.8)	25 (20.8)	1 (3.7)
Peripheral artery disease	55 (10.3)	19 (4.8)	7 (5.8)	1 (3.7)
Stroke/TIA	141 (26.4)	56 (14.1)	36 (30.0)	7 (25.9)
Seizure	12 (2.2)	39 (9.8)	9 (7.5)	1 (3.7)
Functional status at admission				
Premorbid mRS ^a^	1 (0–3)	0 (0–2)	2 (0–3)	1 (0–3)
NIHSS at admission ^b^	3 (1–7)	0 (0–2)	1 (0–3)	2 (0–5)
Prior medication				
Oral anticoagulation	96 (18.0)	51 (13.6)	19 (15.8)	4 (14.8)
Antiplatelet	183 (34.3)	75 (18.9)	36 (30.0)	6 (22.2)

Data are *n* (%) or median (IQR). Abbreviations: mRS, modified Rankin scale; NIHSS, National Institutes of Health Stroke Scale; TIA, transient ischemic attack. ^a^ Data available in *n* = 525 (true positive), *n* = 345 (true negative), *n* = 112 (false positive), and *n* = 27 (false negative); ^b^ data available in *n* = 528 (true positive), *n* = 335 (true negative), *n* = 116 (false positive), and *n* = 26 (false negative).

## Data Availability

Data are available upon reasonable request from the corresponding author.

## Appendix A

**Table A1:** Comparison of patient characteristics of true-positive vs. false-positive initial cerebrovascular diagnoses (*n* = 654).

	True Positive *n* = 534	False Positive *n* = 120	*p*-Value
Age	76.4 (66.1–82.8)	73.2 (60.7–81.0)	**0.012**
Female sex	254 (47.6)	59 (49.2)	0.763
Medical history			
Arterial hypertension	384 (71.9)	61 (51.3)	**<0.001**
Atrial fibrillation	120 (22.5)	27 (22.5)	>0.99
Diabetes mellitus	157 (29.5)	26 (21.7)	0.092
Hyperlipidemia	151 (28.3)	26 (21.7)	0.172
Intracranial hemorrhage	11 (2.1)	4 (3.3)	0.495
Ischemic heart disease	101 (18.9)	25 (20.8)	0.611
Peripheral artery disease	55 (10.3)	7 (5.8)	0.167
Stroke/TIA	141 (26.4)	36 (30.0)	0.428
Seizure	12 (2.2)	9 (7.5)	**0.007**
Functional status at admission			
Premorbid mRS ^a^	1 (0–3)	2 (0–3)	0.542
NIHSS at admission ^b^	3 (1–7)	1 (0–3)	**<0.001**
Prior medication			
Oral anticoagulation	96 (18.0)	19 (15.8)	0.892
Antiplatelet	183 (34.3)	36 (30.0)	0.586

Data are *n* (%) or median (IQR). Abbreviations: mRS, modified Rankin scale; NIHSS, National Institutes of Health Stroke Scale; TIA, transient ischemic attack. ^a^ Data available in *n* = 525 (true positive) and *n* = 112 (false positive); ^b^ data available in *n* = 528 (true positive) and *n* = 116 (false positive).

**Table A2:** Comparison of patient characteristics of true-positive vs. false-negative initial cerebrovascular diagnoses (*n* = 561).

	True Positive *n* = 534	False Negative *n* = 27	*p*-Value
Age	76.4 (66.1–82.8)	78.7 (61.2–85.4)	0.736
Female sex	254 (47.6)	13 (48.1)	>0.99
Medical history			
Arterial hypertension	384 (71.9)	20 (74.1)	>0.99
Atrial fibrillation	120 (22.5)	5 (18.5)	0.814
Diabetes mellitus	157 (29.5)	7 (25.9)	0.830
Hyperlipidemia	151 (28.3)	5 (18.5)	0.379
Intracranial hemorrhage	11 (2.1)	–	>0.99
Ischemic heart disease	101 (18.9)	1 (3.7)	**0.042**
Peripheral artery disease	55 (10.3)	1 (3.7)	0.505
Stroke/TIA	141 (26.4)	7 (25.9)	>0.99
Seizure	12 (2.2)	1 (3.7)	0.477
Functional status at admission			
Premorbid mRS ^a^	1 (0–3)	1 (0–3)	0.935
NIHSS at admission ^b^	3 (1–7)	2 (0–5)	0.072
Prior medication			
Oral anticoagulation	96 (18.0)	4 (14.8)	0.802
Antiplatelet	183 (34.3)	6 (22.2)	0.215

Data are *n* (%) or median (IQR). Abbreviations: mRS, modified Rankin scale; NIHSS, National Institutes of Health Stroke Scale; TIA, transient ischemic attack. ^a^ Data available in *n* = 525 (true positive) and *n* = 27 (false negative); ^b^ data available in *n* = 528 (true positive) and *n* = 26 (false negative).
